# SPINAL DISORDERS IN PROFESSIONAL ESPORTS ATHLETES: PROTOCOL FOR A SYSTEMATIC REVIEW

**DOI:** 10.1590/1413-785220263402e295703

**Published:** 2026-05-11

**Authors:** Newton Yukio Miachiro, Marcelo Diniz de Menezes, Mariana Demétrio de Sousa Pontes, Carlos Fernando Pereira da Silva Herrero

**Affiliations:** 1 Universidade de Sao Paulo, Faculdade de Medicina de Ribeirao Preto (FMRPUSP), Ribeirao Preto, Sao Paulo, Brazil.; 2 Faculdade Pernambucana de Saude (FPS), Recife, Pernambuco, Brazil.

**Keywords:** Spine, Computer Game, Musculoskeletal Pain, Musculoskeletal Diseases, Professional Practice, Coluna Vertebral, Jogos de Computador, Dor Musculoesquelética, Doenças Musculoesqueléticas, Prática Profissional

## Abstract

Electronic sports, or esports, have become increasingly popular worldwide. Official tournaments are organized for participants to compete in a specific gaming titles. However, professional-level competitive participation can lead to health problems that affect the player's quality of life and performance. Previous studies have shown that participation in esports is associated with health risks and challenges, such as a higher incidence of musculoskeletal injuries, sleep problems, sedentary lifestyles, anxiety, and depression. This article presents a systematic review protocol that aims to investigate the effects of professional esports practice on the musculoskeletal system, with a specific focus on the spine. A systematic literature review will be conducted, including observational and intervention studies that evaluate musculoskeletal changes in the spine. This systematic review is expected to provide an overview of the characteristics of professional esports players with spinal complaints and the symptoms observed. The findings of this review may help develop prevention and rehabilitation strategies to promote the physical health of esports players. **Level of Evidence II; Systematic Review**.

## RESUMO

Os esportes eletrônicos (e-sports) têm se tornado cada vez mais populares em todo o mundo. Atualmente, torneios oficiais são organizados para os participantes competirem em determinada modalidade de jogos virtuais. No entanto, a prática profissional pode levar a problemas de saúde que afetam a qualidade de vida e o desempenho do jogador. Estudos prévios demonstraram que a participação em e-sports está associada a riscos e desafios para a saúde, como maior incidência de lesões musculoesqueléticas. Este artigo apresenta um protocolo de revisão sistemática que tem como objetivo investigar os efeitos da prática profissional de esportes eletrônicos no sistema musculoesquelético, com foco específico na coluna vertebral. Será realizada uma revisão sistemática da literatura, na qual serão incluídos estudos observacionais e de intervenção que avaliam queixas musculoesqueléticas da coluna, como dor, incapacidade funcional e alterações de sensibilidade. Espera-se que esta revisão sistemática forneça uma visão geral das características dos jogadores profissionais de e-sports que apresentam queixas na coluna vertebral e dos sintomas observados. Os achados desta revisão podem ser úteis no desenvolvimento de estratégias de prevenção e reabilitação para promover a saúde física destes profissionais. **Nível de Evidência II; Revisão Sistemática**.

## INTRODUCTION

Electronic sports, or e-sports, have grown exponentially in popularity and have established themselves as a form of competition and entertainment widely practiced worldwide.^1^ The intensive practice of electronic games can lead to a series of health problems, which affect players’ quality of life and performance.^1^

In a recently published review, researchers investigated the relationship between participation in e-sports and different aspects of health, including physical health, mental health, cognitive abilities, health behaviors and quality of life.^2^ Although they found positive results related to the improvement of cognitive ability, motor coordination and decision-making in virtual gamers, the authors noted that this participation may be associated with some health risks and challenges, such as a higher risk of developing musculoskeletal injuries, sleep problems, sedentary lifestyle and mental health problems such as anxiety and depression.^2^

Once this relationship between health and the practice of sports has been established, some authors argue that sports medicine should expand its focus to include virtual sports, providing guidance and support to practitioners.^3^ They highlight the importance of a multidisciplinary approach involving physicians, physical therapists, nutritionists, and psychologists to assess and address the specific health needs of esports players.^3^

Data indicate that 76% of college e-sports players reported at least one musculoskeletal injury during their competitive career.^4^ Among the problems identified, pain in the lower back, neck, shoulders, and hands, as well as symptoms of muscle fatigue and stiffness, stand out.^5^ These researchers suggest that e-sports players are susceptible to developing musculoskeletal disorders, possibly due to factors such as poor posture during play, repetitive movements, and lack of adequate intervals.^5^

In an update on e-sports medicine focusing on pre-participation screening and injury management, the authors discuss the most common dysfunctions and highlight carpal tunnel syndrome, tendinopathies, and spinal injuries as the primary musculoskeletal injuries.^6^ This study also highlights the importance of adequate rehabilitation and a gradual return to training and competition after an injury.^6^

Recent reports in the literature have shown that electronic sports can negatively influence the musculoskeletal system, affecting different body regions, including the spine.^4-6^ How professional practice influences this condition has yet to be discussed. This systematic review aims to provide an overview and review the evidence available in the literature regarding how the professional practice of electronic sports (e-sports) interferes with musculoskeletal conditions of the spine. So, the following question will be investigated: What are the characteristics of musculoskeletal injuries of the spine presented by professional electronic sports players?

The findings of this review may provide relevant information for developing prevention and rehabilitation strategies that favor the physical health of professional electronic sports players.

## METHODS

### Research Question

This article will aim to answer the question, "How are musculoskeletal injuries of the spine presented by professional e-sports players characterized?" which was developed using the acronym PICO.^7^ In this context, "P" represents the population involved in the research, "I" refers to the intervention or exposure studied, "C" refers to the control used, and "O" encompasses the outcomes analyzed.^7^

The population included in this study consists of professional e-sports players over the age of 15. The intervention will be based on the professional practice of e-sports using computers. The control will be represented by people who do not practice electronic games using computers. The outcome will be assessed through musculoskeletal injuries of the spine, such as pain, functional disability and changes in sensitivity originating in the spine.

### Eligibility criteria

Observational studies (case reports, case series, cross-sectional, case-control, cohort) and intervention studies (quasi-experimental studies, randomized controlled trials, community trials, field trials) that assess outcomes related to musculoskeletal injuries of the spine, such as pain, functional disability, and changes in strength or sensitivity in the limbs will be included. The study sample must be composed of professional e-sports players of both sexes who use computers aged 15 or older. Studies in which players use other tools, such as racing simulators (steering wheel or bicycle), cell phones, and console controllers, will be excluded, as well as studies that assess only psychological, social, or cardiovascular outcomes.

### Sources of information

The following databases will be consulted: MEDLINE (via PubMed), Embase, PEDro, LILACS, SciELO, Scopus, and Web of Science Cochrane Library. There will be no language or year of publication limit. In addition to the terms belonging to the Medical Subject Headings (MeSH),^8^ words related to the professional practice of electronic sports and musculoskeletal injury or pain will be used to identify appropriate keywords.

### Search strategy

The terms used in the search will be: e-sport, e-sports, esport and esports. These terms will be worked with the Boolean operators [OR] and [AND] in advanced searches. Two researchers will search independently, following the Peer Review of Electronic Search Strategies (PRESS) protocol rules.^9^

### Data recording

Two researchers will carry out the selection of studies that will be part of the systematic review in two stages. In the first phase, the evaluators will select potentially relevant studies based on the titles and abstracts. The full text of the studies selected in the previous phase will be read in the second phase. The articles chosen for full reading will be evaluated according to the eligibility criteria.

If opinions are divergent, the impasse will be resolved through consensus with a third evaluator specialized in the subject. In addition, Mendeley (Mendeley, London, United Kingdom), a reference manager available as an online application, will be used to avoid duplication and facilitate file sharing between researchers.

The two evaluators will also extract the data from the studies in a paired and independent manner. The systematic review manager Rayyan (Rayyan Systems Inc., Cambridge, MA, USA), available in the form of a web application, will be used to allow better exploration of the results of the included studies. The third researcher will analyze possible disagreements.

The data extraction form will contain the following:

- general information: title, authors, year of publication, study design, country of origin.
- characteristics of participants: age, sex, length of professional experience, physical activity practice and body mass index (BMI).
- study design.
- results: number of participants, measurement tools and respective results (numerical pain scale, self-report questionnaires, dynamometry, endurance tests), location of symptoms, statistical tests, descriptive information (type of console, daily practice time).

### Analysis of the Risk of Bias of the Included Studies

An adapted version of the Newcastle-Ottawa Scale (NOS)^10^ will be used to analyze the risk of bias of the studies included in the review for cross-sectional studies and the ROB 2.0 tool for intervention studies. The NOS scale includes four items for selection, one for comparability and two for outcome. Each study can be rated on a scale of 0 to 10 stars, with a total of 7 or more stars indicating high quality, 4 to 6 stars indicating medium quality, and 3 or fewer stars indicating low quality. The ROB 2.0 tool assesses the risk of bias in intervention studies in five domains: bias resulting from the randomization process, bias related to deviations in the application of interventions, bias due to missing outcome data, bias in the measurement of outcomes, and bias in the selection of reported outcomes.

### Data synthesis

The studies found are expected to be heterogeneous in terms of study design and description of results. Therefore, the results will be summarized in a narrative format, without additional statistical analysis.

### Assessment of the Quality of Evidence

After completing the critical evaluation of the study methodology, the quality of the evidence related to each outcome assessed in the systematic review will be carefully considered. The Grading of Recommendations Assessment, Development, and Evaluation (GRADE) system will be used to classify the level of evidence for each outcome as high, moderate, low, or very low.^11^

### Registration

This protocol was registered on the PROSPERO platform^12^ (CRD42024527224) according to the guidelines for systematic reviews of the Centre for Reviews and Dissemination (CRD) at the University of York.^13^ The results of the review will be reported following the PRISMA guidelines.^14^ As this work will be based on published studies, there is no need for approval by the ethics committee.

### Amendments

This protocol does not imply a modification of a previously completed or published protocol. If necessary, any amendments to the protocol will also be registered on the PROSPERO platform.^12^

## Data Availability

The data will be made available when requested.
